# Supplementary material for: Monitoring Appropriate Monoclonal Antibodies Prescribing via Administrative Data: An Application to Psoriasis
Source: Pharmaceuticals (Basel). 2022 Oct 8;15(10):1238. doi: 10.3390/ph15101238 (PMC9610535; doi:10.3390/ph15101238)
Supplement: Supplementary file 1 [file pharmaceuticals-15-01238-s001.zip › pharmaceuticals-1921627-supplementary.pdf]

Table S1

	First supply, line of treatment	Last supply, line of treatment	Number of patients	% over the total per AV
North-West AV	1st	1st	1079	54.22%
North-West AV	1st	2nd	237	11.91%
North-West AV	2nd	1st	44	2.21%
North-West AV	2nd	2nd	630	31.66%
Centre AV	1st	1st	1370	60.92%
Centre AV	1st	2nd	197	8.76%
Centre AV	2nd	1st	62	2.76%
Centre AV	2nd	2nd	620	27.57%
South-East AV	1st	1st	742	49.34%
South-East AV	1st	2nd	174	11.57%
South-East AV	2nd	1st	45	2.99%
South-East AV	2nd	2nd	543	36.10%

Table S2

	Number of patients treated in 2020 with second-line mAbs	Cohort of 2020 psoriatic patients	% Over the total cohort
	**Adalimumab**		
**Tuscany region**	634	5743	11.04%
North-West AV	267	1990	13.42%
Centre AV	187	2249	8.31%
South-East AV	180	1504	11.96%
	**Etanercept**		
**Tuscany region**	412	5743	7.17%
North-West AV	150	1990	7.54%
Centre AV	150	2249	6.69%
South-East AV	112	1504	7.45%
	**Sekukinumab**		
**Tuscany region**	285	5743	4.96%
North-West AV	97	1990	4.87%
Centre AV	97	2249	4.31%
South-East AV	91	1504	6.05%
	**Ustekinumab**		
**Tuscany region**	259	5743	4.50%
North-West AV	97	1990	4.87%
Centre AV	80	2249	3.55%
South-East AV	82	1504	5.45%
	**Ixekizumab**		
**Tuscany region**	136	5743	2.37%
North-West AV	53	1990	2.66%
Centre AV	43	2249	1.91%
South-East AV	40	1504	2.66%
	**Infliximab**		
**Tuscany region**	97	5743	1.69%
North-West AV	35	1990	1.76%
Centre AV	57	2249	2.53%
South-East AV	5	1504	0.33%
	**Guselkumab**		
**Tuscany region**	94	5743	1.64%
North-West AV	39	1990	1.96%
Centre AV	29	2249	1.29%
South-East AV	26	1504	1.73%
	**Certolizumab**		
**Tuscany region**	59	5743	1.03%
North-West AV	14	1990	0.70%
Centre AV	27	2249	1.20%
South-East AV	18	1504	1.19%

Table S3

	Adalimumab		
	**Number of psoriatic patients treated in 2020 with Adalimumab that received as first supply a first-line treatment**	**Number of psoriatic patients treated in 2020 with Adalimumab**	**%**
**Tuscany region**	165	634	26.02%
North-West AV	73	267	27.34%
Centre AV	43	187	22.99%
South-East AV	49	180	27.22%
	**Number of psoriatic patients treated in 2020 with Adalimumab that received Adalimumab as first supply**	**Number of psoriatic patients treated in 2020 with Adalimumab**	**%**
**Tuscany region**	407	634	64.19%
North-West AV	175	267	65.54%
Centre AV	123	187	65.77%
South-East AV	109	180	60.55%
	**Number of psoriatic patients treated in 2020 with Adalimumab that received a second-line treatment as first supply**	**Number of psoriatic patients treated in 2020 with Adalimumab**	**%**
**Tuscany region**	62	634	9.77%
North-West AV	19	267	7.11%
Centre AV	21	187	11.22%
South-East AV	22	180	12.22%
	**Etanercept**		
	**Number of psoriatic patients treated in 2020 with Etanercept that received as first supply a first-line treatment**	**Number of psoriatic patients treated in 2020 with Etanercept**	**%**
**Tuscany region**	131	412	31.79%
North-West AV	51	150	34.00%
Centre AV	49	150	32.67%
South-East AV	31	112	27.68%
	**Number of psoriatic patients treated in 2020 with Etanercept that received Etanercept as first supply**	**Number of psoriatic patients treated in 2020 with Etanercept**	**%**
**Tuscany region**	255	412	61.89%
North-West AV	92	150	61.33%
Centre AV	93	150	62.00%
South-East AV	70	112	62.50%
	**Number of psoriatic patients treated in 2020 with Etanercept that received a second-line treatment as first supply**	**Number of psoriatic patients treated in 2020 with Etanercept**	**%**
**Tuscany region**	26	412	6.31%
North-West AV	7	150	4.67%
Centre AV	8	150	5.33%
South-East AV	11	112	9.82%

Certolizumab			
	**Number of psoriatic patients treated in 2020 with Certolizumab that received as first supply a first-line treatment**	**Number of psoriatic patients treated in 2020 with Certolizumab**	**%**
**Tuscany region**	9	59	15.25%
North-West AV	4	14	28.57%
Centre AV	3	27	11.11%
South-East AV	2	18	11.11%
	**Number of psoriatic patients treated in 2020 with Certolizumab that received Certolizumab as first supply**	**Number of psoriatic patients treated in 2020 with Certolizumab**	**%**
**Tuscany region**	24	59	40.67%
North-West AV	4	14	28.57%
Centre AV	16	27	59.25%
South-East AV	4	18	22.22%
	**Number of psoriatic patients treated in 2020 with Certolizumab that received a second-line treatment as first supply**	**Number of psoriatic patients treated in 2020 with Certolizumab**	**%**
**Tuscany region**	26	59	44.06%
North-West AV	6	14	42.85%
Centre AV	8	27	29.62%
South-East AV	12	18	66.66%

Infliximab			
	**Number of psoriatic patients treated in 2020 with Infliximab that received as first supply a first-line treatment**	**Number of psoriatic patients treated in 2020 with Infliximab**	**%**
**Tuscany region**	4	97	4.12%
North-West AV	0	35	0
Centre AV	4	57	7.02%
South-East AV	0	5	0
	**Number of psoriatic patients treated in 2020 with Infliximab that received Infliximab as first supply**	**Number of psoriatic patients treated in 2020 with Infliximab**	**%**
**Tuscany region**	75	97	77.31%
North-West AV	30	35	85.71%
Centre AV	41	57	71.93%
South-East AV	4	5	80.00%
	**Number of psoriatic patients treated in 2020 with Infliximab that received a second-line treatment as first supply**	**Number of psoriatic patients treated in 2020 with Infliximab**	**%**
**Tuscany region**	18	97	18.55%
North-West AV	5	35	14.28%
Centre AV	12	57	21.05%
South-East AV	1	5	20.00%

	Sekukinumab		
	**Number of psoriatic patients treated in 2020 with Sekukinumab that received as first supply a first-line treatment**	**Number of psoriatic patients treated in 2020 with Sekukinumab**	**%**
**Tuscany region**	88	285	30.87%
North-West AV	28	97	28.86%
Centre AV	30	97	30.92%
South-East AV	30	91	32.96%
	**Number of psoriatic patients treated in 2020 with Sekukinumab that received Sekukinumab as first supply**	**Number of psoriatic patients treated in 2020 with Sekukinumab**	**%**
**Tuscany region**	72	285	25.26%
North-West AV	20	97	20.61%
Centre AV	25	97	25.77%
South-East AV	27	91	29.67%
	**Number of psoriatic patients treated in 2020 with Sekukinumab that received a second-line treatment as first supply**	**Number of psoriatic patients treated in 2020 with Sekukinumab**	**%**
**Tuscany region**	125	285	43.85%
North-West AV	49	97	50.51%
Centre AV	42	97	43.29%
South-East AV	34	91	37.36%
	**Ustekinumab**		
	**Number of psoriatic patients treated in 2020 with Ustekinumab that received as first supply a first-line treatment**	**Number of psoriatic patients treated in 2020 with Ustekinumab**	**%**
**Tuscany region**	52	259	20.07%
North-West AV	22	97	22.68%
Centre AV	17	80	21.25%
South-East AV	13	82	15.85%
	**Number of psoriatic patients treated in 2020 with Ustekinumab that received Ustekinumab as first supply**	**Number of psoriatic patients treated in 2020 with Ustekinumab**	**%**
**Tuscany region**	145	259	55.98%
North-West AV	53	97	54.63%
Centre AV	39	80	48.75%
South-East AV	53	82	64.63%
	**Number of psoriatic patients treated in 2020 with Ustekinumab that received a second-line treatment as first supply**	**Number of psoriatic patients treated in 2020 with Ustekinumab**	**%**
**Tuscany region**	62	259	23.93%
North-West AV	22	97	22.68%
Centre AV	24	80	30.00%
South-East AV	16	82	19.51%

	Guselkumab		
	**Number of psoriatic patients treated in 2020 with Guselkumab that received as first supply a first-line treatment**	**Number of psoriatic patients treated in 2020 with Guselkumab**	**%**
**Tuscany region**	38	94	40.42%
North-West AV	17	39	43.58%
Centre AV	12	29	41.37%
South-East AV	9	26	34.61%
	**Number of psoriatic patients treated in 2020 with Guselkumab that received Guselkumab as first supply**	**Number of psoriatic patients treated in 2020 with Guselkumab**	**%**
**Tuscany region**	14	94	14.89%
North-West AV	9	39	23.07%
Centre AV	1	29	3.44%
South-East AV	4	26	15.38%
	**Number of psoriatic patients treated in 2020 with Guselkumab that received a second-line treatment as first supply**	**Number of psoriatic patients treated in 2020 with Guselkumab**	**%**
**Tuscany region**	42	94	44.68%
North-West AV	13	39	33.33%
Centre AV	16	29	55.17%
South-East AV	13	26	50.00%
	**Ixekizumab**		
	**Number of psoriatic patients treated in 2020 with Ixekizumab that received as first supply a first-line treatment**	**Number of psoriatic patients treated in 2020 with Ixekizumab**	**%**
**Tuscany region**	61	136	44.85%
North-West AV	24	53	45.28%
Centre AV	17	43	39.53%
South-East AV	20	40	50.00%
	**Number of psoriatic patients treated in 2020 with Ixekizumab that received Ixekizumab as first supply**	**Number of psoriatic patients treated in 2020 with Ixekizumab**	**%**
**Tuscany region**	12	136	8.82%
North-West AV	5	53	9.43%
Centre AV	2	43	4.65%
South-East AV	5	40	12.50%
	**Number of psoriatic patients treated in 2020 with Ixekizumab that received a second-line treatment as first supply**	**Number of psoriatic patients treated in 2020 with Ixekizumab**	**%**
**Tuscany region**	63	136	46.32%
North-West AV	24	53	45.28%
Centre AV	24	43	55.81%
South-East AV	15	40	37.50%
